# The mechanism for microsporidian parasite suppression of the hindgut bacteria of the migratory locust *Locusta migratoria manilensis*

**DOI:** 10.1038/srep17365

**Published:** 2015-11-27

**Authors:** Shu-qian Tan, Kai-qi Zhang, Hong-xing Chen, Yang Ge, Rong Ji, Wang-peng Shi

**Affiliations:** 1 Department of Entomology, China Agricultural University, Beijing, China; 2 College of Life Science, Xinjiang Normal University, Urumchi, China

## Abstract

Locusts aggregate into bands of nymphs and swarms of adults that can pose a major threat to crop. Previous studies have shown that infection by the microsporidian parasite *Paranosema locustae* prevents locust aggregation behavior and we show that gut bacteria, which produce components of locust aggregation pheromones, are substantially reduced in locusts infected with *P. locustae*. We found that *P. locustae* could reduce the diversity, abundance and community composition of *Locusta migratoria*’s gut bacteria. The parasite infection was also shown to interrupt the peroxidase activity of locust hindgut. Genome-wide expression analysis showed that the parasite infection suppressed *peroxidase* mRNA relative expression of locust hindgut, but had no effects on *attacin* expression and superoxide dismutase at 16 d post-inoculation with 20,000 *P. locustae* spores. Our findings reveal the mechanisms by which *P. locustae* impairs bacterial diversity and community structure of *Locusta migratoria*’s gut bacteria.

*Paranosema locustae* Canning is a common entomopathogen that can control populations of grasshoppers with long-term effectiveness. Some studies have demonstrated that *P. locustae* remained present in grasshopper populations for many years after application^1^,^2^. Populations of nymphs were reduced significantly when *Locusta migratoria manilensis* were sprayed with *P. locustae* and, some time before mortality was evident, crowded groups fragmented into many smaller ones. This fragmentation was the result of aggregation pheromone production being disrupted in locusts infected by *P. locustae* such that their aggregation behaviour was weakened in fourth and fifth instar nymphs^3^,^4^,^5^.

Hundreds of species of bacteria live in the insect gut; these bacteria contribute to the behavior, physiology and immunity of insect, aid digestion and synthesis of vitamins, improvement of the absorption of carbon and nitrogen nutrition, modulation of insect immunity system, and are an important source of functioning enzymes, such as cellulase, xylanase etc^6^. In addition, locust aggregation relates to aggregation pheromones, and some components of these pheromones are produced by gut bacteria^7^,^8^. Recently, Chinese scientists provided new proofs for the change in locust behavioral change being a result of reduced levels of the aggregation pheromones after spraying of *P. locustae*^5^. So, the relationship between gut bacteria and *P. locustae* is an essential link for understanding locust behavior.

It is well understood that the host’s development stages, diet and the habitat it occupies influence the constitution of its gut bacteria community^9^,^10^,^11^,^12^. However, exogenous microorganism colonization can also influence intestinal bacteria communities^13^. From the standpoint of both basic researches, there is considerable interest in reaching a clearer understanding of the diversity transformation of intestinal microbiota of locusts after infection with the introduced microsporidian *P. locustae*. The aim of the present study was to determine how *P. locustae* alters the host locust’s gut microbiota. We demonstrate that this parasite reduces bacterial abundance and diversity in the hindgut of infected locusts by inhibiting peroxidase (POD) activity and increasing the Reactive Oxygen Species (ROS) in the locust gut.

## Results

### The bacterial abundance in the hindgut decreased in locusts infected with Paranosema locustae

There were 49515 raw reads sequenced with Miseq and the average length was 301 bp. Then, the chimera and Achaea sequences were checked and removed. The number of effective bacterial sequences obtained for infected locust (IL) samples were less than those of control locusts (CL), but the average length was not significantly different (Table 1). Almost all (99.95%) sequences were between 300 and 400 bp, with very few at <300 bp or >400 bp.

**Figure 1:**
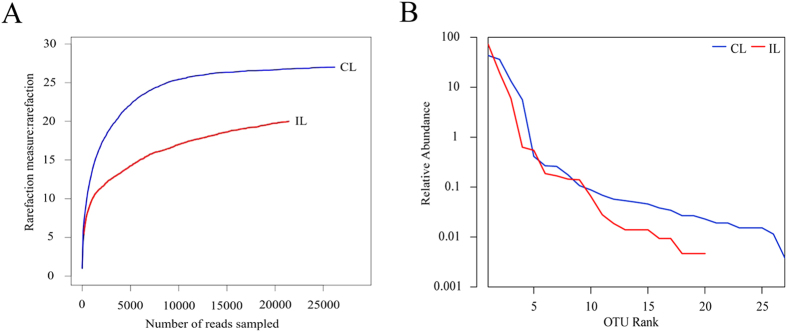
(**A**) Rarefaction curves; (**B**) Abundance and uniformity are shown, rank-abundance distribution curve. Red line: infected locust (IL); blue one: control locust (CL).

The rationalisation of sampling was described by a Rarefaction curve (Fig. 1A). The Rarefaction curve showed that the numbers of bacterial Operational Taxonomic Units (OTUs) approached a certain value, leveling off at a high sampling size, which meant that the number of bacterial sequences obtained were reasonable and the bacterial diversity of the samples were accurate, The rank-abundance distribution curve showed that the bacterial abundance in the hindgut of infected locusts was lower than that for untreated control locusts (Fig. 1B). The rank abundance curve shows that the more common bacteria were still present in infected locusts, but many of the less common bacteria were absent (Fig. 1B).

### *Paranosema locustae* colonies impaired the bacterial diversity of migratory locust’s gut

The diversity of bacterial communities of treated and untreated locusts was analyzed using the Alpha diversity method. The Ace and Chao indices were used for estimating community richness; while the Shannon and Simpson indices were applied for estimating community diversity; four indices were obtained with the Mothur software package^14^. The indices showed that both bacterial abundance and diversity were lower in the hindgut of infected locusts than in the untreated controls (Table 2). Furthermore, more bacterial communities were living in gut of healthy locusts (Table 2). Overall then, there was a dramatic simplification of the hindgut microbiota communities’ structure in locusts infected by *P. locustae*. Moreover, the diversity of bacterial communities of wild locusts was analyzed and the results showed that both bacterial abundance and diversity were higher in the wild locusts than in the laboratory strains (Table 2 and Table S3).

### *Paranosema locustae* reduced the hindgut bacterial community composition of locusts

**Figure 2:**
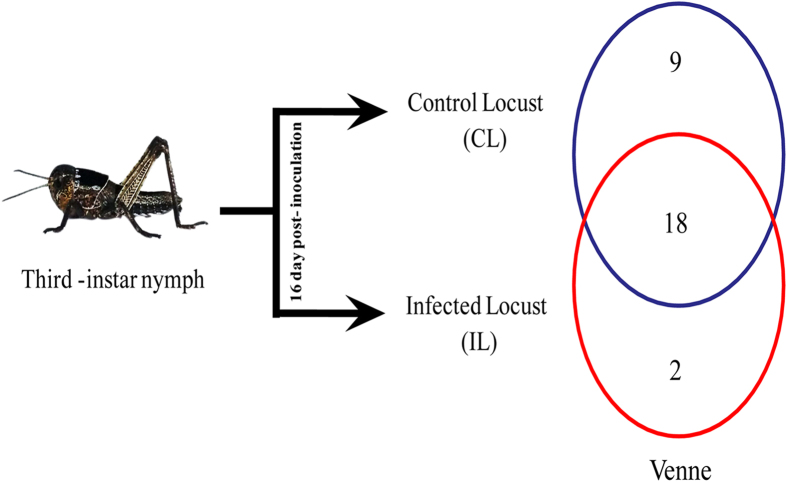
Venne diagram of Bacteria Operational Taxonomic Units. red = infected locusts (IL), blue = control locusts (CL). The picture in this figure was a photo which was taken and modified by S.-Q.T.

**Figure 3:**
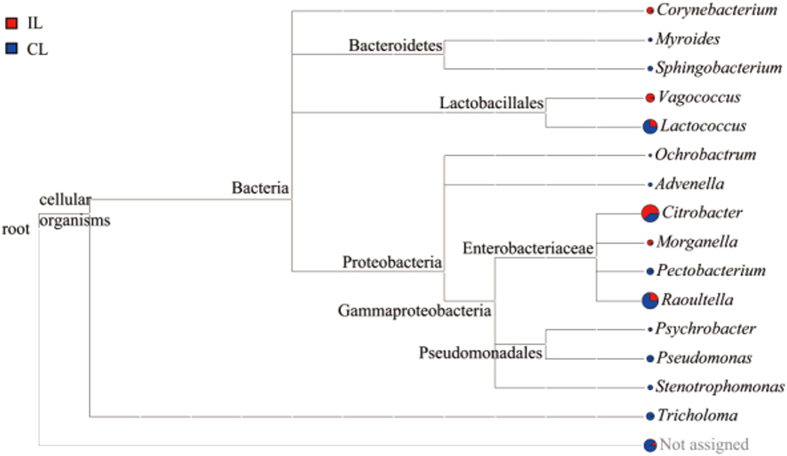
Bacterial genera identified in the locust hindgut. Bacteria were classified according to branching in the 16 S rRNA sequence phylogenetic tree. Sequences assignment results at the genus level. *Tricholoma* is one genus of fungus. red = infected locusts (IL), blue = control locusts (CL). Many other bacteria were not identified were included in “Not assigned”.

Using a minimum of 97% of a sequence being identical as the threshold for any sequence pair being the same operational taxonomic units (OTUs), we identified 29 different bacterial OTUs in the laboratory colony, but 50 OTUs in the wild locusts (Table 2 and Table S3). The Venne diagram (Fig. 2) showed that of the 29 OTUs detected, 9 were found only in uninfected controls, 2 were only in infected locusts and 18 were in both. Overall, there were 27 OTUs detected in the control locusts, but only 20 in infected locusts. The details of the bacterial genera found are shown in Fig. 3.

**Figure 4:**
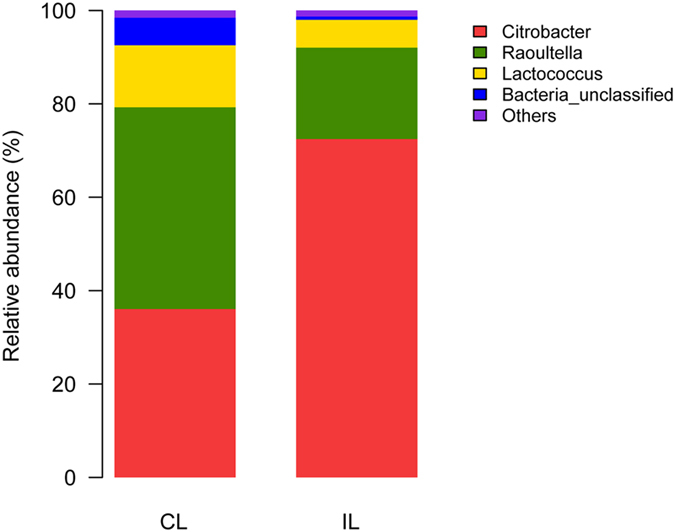
Relative abundance of genus level classified for two samples: CL = control locusts, IL = infected locusts.

The Ribosomal Database Project Classifier software used 16s RNA sequence data to classify the OTUs: those with a 97% similarity level were classified into the same taxonomic group. And the bacterial communities (laboratory colony) were estimated in different taxonomic levels belonged to 5 phyla, 8 Classes, 10 orders, 12 families, 15 genera and 15 species. The taxonomic tree (Fig. 3) suggested that 1 fungal (*Tricholoma*) and 14 bacterial genera examined from control locusts were *Advenella, Citrobacter, Corynebacterium, Lactococcus, Morganella, Myroides, Ochrobactrum, Pectobacterium, Pseudochrobactrum, Psychrobacter, Raoultella, Sphingobacterium, Stenotrophomonas* and *Vagococcus*. Clones belonging to *Citrobacter* (36.08%), *Lactococcus* (13.28%) and *Raoultella* (43.2%) were the most predominant, but *Citrobacter* (72.48%) and *Raoultella* (19.56%) were mainly found in infected locusts (Fig. 4). Moreover, *Bacillus* and *Pseudomonas* were mainly genera in the wild strain (Fig. S2). There were 10 genera of bacteria present in infected locusts, but 4 genera were absent: *Advenella, Ochrobactrum, Sphingobacterium* and *Stenotrophomonas*. The richness of 10 genera decreased after infection.

High-throughput pyrosequencing showed that approximately 51.7% of the OTUs in the hindgut microenvironment can be identified at species level here, the details of identification were showed in (Table S2). Only 1 species, *Corynebacterium sp. WA7*, increased in infected locusts, but 7 species reduced and 7 species disappeared. The main species, *Raoultella (Klebsiella) terrigena*, had reduced by 50% more. Despite all this, there were a number of hindgut bacteria (6 genera and 2 species) which were found both in the laboratory strains and the wild locusts (Fig. 3 and Fig. S1; Table S2 and Table S4).

### Infected locusts have reduced peroxidase activity in their hindguts, but there is no effect on superoxide dismutase

**Figure 5:**
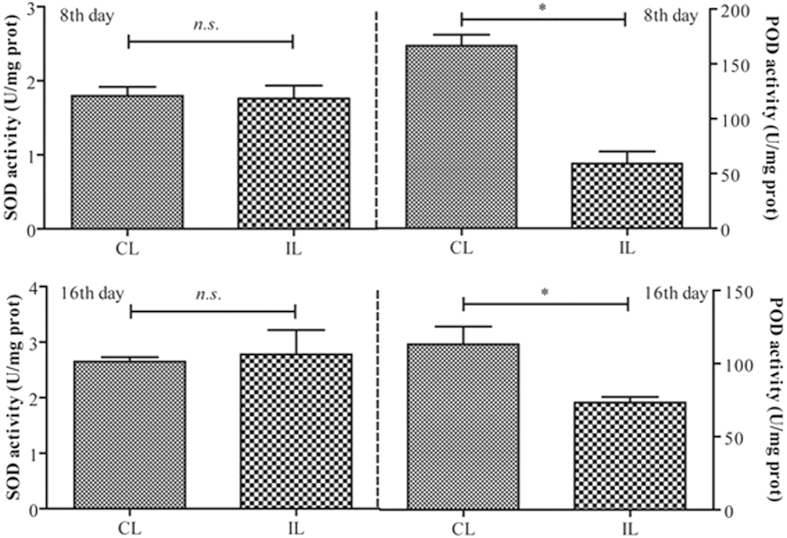
Superoxide dismutase (SOD) and peroxidase (POD) activity (mean ± SE) of *L. migratoria* hindgut, 8 & 16 days after inoculation with *P. locustae*. CL: control locusts; IL: locusts infected with 20,000 *P. locustae* spores. Values with th*e* “*n.s.*” are not significantly different (P > 0.05) but with the “*” are significantly different (P ≤ 0.05) (Independent-Samples T test).

Superoxide dismutase (SOD) is an enzyme that can transform reactive oxygen species into peroxide in cells. And peroxidase (POD) can eliminate peroxide. In the study, the activity of SOD and POD of locust hindguts were measured at 8 and 16 d after inoculation with *P. locustae*. The results showed that the values of SOD activity were unchanged significantly (P > 0.05), but the values of POD activity were significantly reduced at both 8 and 16 d after inoculation (P ≤ 0.05) (Fig. 5).

### The effects of infection by *P. locustae* on the relative expression *peroxidase* mRNA and attacin

**Figure 6:**
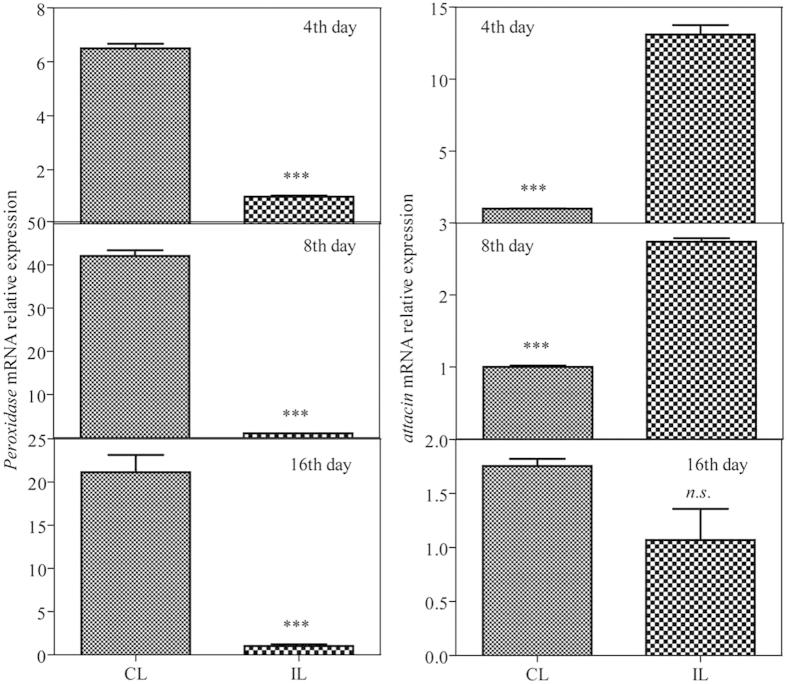
*Peroxidase* and *attacin* mRNA relative expression in samples of *L. migratoria* hindgut at 4, 8 & 16 d after inoculation with *P. locustae*. CL: control locusts; IL: locusts infected with 20,000 *P. locustae* spores. Values with th*e* “*n.s.*” are not significantly different (P > 0.05) but with “***” are significantly different (P ≤ 0.001) (Independent-Samples T test). Data was presented as mean (±SE).

*Peroxidase* mRNA relative expression of locust hindgut was examined using genome-wide expression analysis. The values of expression at 4, 8 and 16 d after infection were reduced 6.5-fold, 42-fold and 20.4-fold, respectively (P ≤ 0.001) (Fig. 6). *Attacin* expression was higher at 4 and 8 d after infection, having increased 13.1-fold and 2.7-fold, respectively (P ≤ 0.001). However, there was no significantly difference at 16 d after infection (P > 0.05).

## Discussion

Intestinal bacteria play an important role in the host’s digestion function and immune reaction, and even on behavior^15^,^16^,^17^,^18^. For example, *Pantoea agglomerans*, a general intestinal bacterium, produced antimicrobial phenols against gut infection^19^. The diversity of the microbiota relates in part to the variety of specialized structures present in the gut and the effects of pH, redox conditions, digestive enzymes present, and type of food ingested: changes in the internal and external conditions of gut, leads to alteration of the host’s intestinal bacteria communities^20^. Bacteria colonized in hindgut of wild locusts and lab colony were different because of the difference of food ingested and enviroment lived. And the wild environment made higher bacterial abundance and diversity. Here we found that the invasion of *P. locustae* changed the community structure of the gut microbes of locusts. *P. locustae* could acidify the hindgut, which contributed to the changes in gut bacteria seen here. At 16 d after inoculation with *P. locustae*, the *L. migratoria* hindgut was more acidic, with the pH declining to 5.6: it is likely that this increased acidity contributed to the general reduction in gut bacteria seen here, as more acidic conditions are less than optimum for most bacteria growth^5^,^20^. Here, two immunogenes’ expression quantity were examined by qRT-PCR, and the results showed that immune response appeared in hindgut because of infection with 20,000 *P. locustae* spores, and that immune response started quite early—by 4 d after inoculation. Moreover, the enzyme assay gave more evidence that peroxide would take part in immunity to general hindgut bacteria.

Our results showed that the diversity of the bacterial community of infected locusts was lower than that of untreated locusts. The two of main genera present in the guts of uninfected locusts, *Lactococcus* and *Raoultella,* were in much lower proportions in infected locusts. The main species *Raoultella terrigena* reduced by 50% more post-treatment with the pathogen. Rats colonized by *R. terrigena* showed greater melamine-induced kidney damage compared to those not colonized, *R. terrigena* in the rat gut had the capacity for converting melamine to cyanuric acid directly^21^. The function of *R. terrigena* in the locust gut is not determined, possiblly relevant to phase transformation and immunity of locust, which need to research further in the future. In nature, the numerous intestinal bacteria constitute a unified micro-ecosystem, which play an important role in the locusts’ characteristic aggregation behavior. Conventionally, the ecosystem whose species abundance is lower is more changeable and can be eliminated more easily. In turn, alien species are also able to more easily colonize an unstable ecosystem^5^,^22^. In this study, the diversity of intestinal bacteria declined after inoculating *P. locustae* of 2 × 10^4^ spores/locust. Locust intestines would be more easily invaded and colonized by other pathogens because of the weakened colonization resistance, which is partially related to the suppression of peroxidase activity in the hindgut of infected locusts. Reduced resistance could contribute to locust mortality from *P. locustae* in the field, which would contribute to exploitation of this microbial pesticide as a biological control product^23^. The competition mechanisms between migratory locust’s gut natural bacterial communities and invading microsporidian parasites need to be examined in further studies.

Infected locusts have reduced peroxidase activity in their hindguts, and the infection by *P. locustae* affected significantly the relative expression peroxidase mRNA and *attacin*, which was a potential mechanism that *P. locustae* reduced the hindgut bacterial community composition of locusts. Because the attacin and reactive oxygen species (ROS) are broad-spectrum bacteriocidal substances which can effectively reduce population of bacteria. And ROS generated are required for limiting the proliferation of local pathogens during gut-microbe interactions^24^. As a major defense of the *Drosophila* gut, it was proved that oxidative burst dramatically increased bacterial infections when epithelium renewal increased in *Drosophila* gut^25^. Taken together, activity of the two substances (attacin and ROS) were potential factors for making microorganism reduce or disappear.

## Materials and Methods

### Insects and infection

Oriental migratory locusts, *Locusta migratoria manilensis* used for the study, were from a laboratory colony originating from a stock obtained for the Key Bio-control Laboratory for Locusts in Beijing, China. Groups of 80–100 hatchlings were reared in a metallic cage (50 cm high by 15 cm diameter) in the lab under a long-day photoperiod (16 h light/8 h darkness cycle) at 30 ± 2 °C and 70% relative humidity. All conditions were controlled by an artificial climate cabinet (PXZ-430B, Ningbo Jiangnan Instrument Factory, China). Nymphs were fed with wheat seedlings. The wild locusts were gathered from Dagang district, Tianjin, China, *Phragmites australis* as main vegetation.

The original stock of *P. locustae* was obtained from the US Department of Agriculture, Agricultural Research and Service, Rangeland Insect Laboratory, Montana State University, Bozeman. *P. locustae* used in this experiment were propagated by the experimental factory at China Agricultural University, Beijing, China. Third instar nymphs were starved for 24 h and each was inoculated with 2 × 10^4^ *P. locustae* spores. Cages of inoculated and of untreated nymphs (controls) were then kept in conditions described above.

### Preparation of locust hindguts

Fifteen days later, both inoculated and untreated nymphs were starved for 24 h. On day 16, hindguts were dissected and collected into centrifugal tube with PBS (PH = 7.4). There were 8–10 hindguts in each centrifugal tube. For extraction of mRNA, the hindguts were collected into 1.5 mL centrifugal tube with 100 μL Trizol. There were 3 replicates of 3 to 5 individuals per replicate.

### DNA extraction and PCR amplification

The DNA extraction and PCR amplification methods used in this paper were as previously described^26^. Total bacterial DNA from hindgut was extracted by E.Z.N.A.^®^ Soil DNA Kit (OMEGA, Norcross, America) and stored at −20 °C until processed. The extracted DNA was then confirmed by 1% agarose gel electrophoresis. The DNA of each sample was then amplified using the special primers (Table S1) targeting the hypervariable V4–V5 region of the bacterial 16S rRNA gene before pyrosequencing^27^. The PCR amplification was conducted in a 20 mL reaction system using TransGen AP221-02: TransStart Fastpfu DNA Polymerase (TransGen Biotech, Beijing, China) and performed in an ABI GeneAmp® 9700 (ABI, Carlsbad, USA) under the following conditions: 95 °C for 1 min, 25 cycles at 94 °C for 30 s, 55 °C for 30 s and 72 °C for 30 s, and a final extension at 72 °C for 5 min, 10 °C. The PCR products were purified using AxyPrep DNA Gel Extracion Kit (AXYGEN, Union City of California, USA) and then were quantitated by QuantiFluor™-ST (Promega, Madison, USA) then mixed equally before pyrosequencing.

### High-throughput pyrosequencing

The PCR products of the V4–V5 region of bacterial 16S rRNA gene were sequenced using the Roche 454 FLX Titanium sequencer (Roche, Nutley, NJ, USA). The samples were individually barcoded to enable multiplex sequencing.

### Sequence processing and bacterial population analysis

Sequencing noises were filtered out by the Precluster tool in the Mothur package^28^. Those being assigned to any known genus with 70% confidence threshold were merged with the non-chimera reads to create the collection of “effective sequences” for each sample. Ribosomal Database Project (RDP) Classifier software^29^ was used to classify the sequences and barcodes were searched from the NCBI database. The rarefaction curves were created using the “RDP Rarefaction” tool. And the effective sequences were then assigned to NCBI taxonomies with molecular evolutionary genetics analysis (MEGAN)^30^.

### Immunity genes qRT-pcr assaying

Three biological replicates (3 to 5 individuals per replicate) were pooled for three parallel technical analyses. The mRNA of each sample was extracted from the dissected hindgut using Invitrogen TRIzol Reagent (Life technologies, USA). The first-strand cDNA was synthesized using FastQuant RT Kit (with gDNase) (TIANGEN BIOTHECH, China). The cDNA was serially diluted 5-fold and the dilutions were used for analyzing PCR efficiency of primers. The preparation of RT- PCR mixture was conducted as instructed by the manual of the SYBR® Premix Ex TaqTM (Tli RNaseH Plus) (Takara, China). The reactions were performed on a StepOne (ABI, USA) using the two-step method, and completed with a melting curve analysis program. The specificity of qRT-PCR primers was confirmed by melting curve and sequencing of qRT-PCR products. The actin sequence of *L. migratoria* (GenBank accession no: AF370793) were used as the internal control. All primers are presented in Table S1. Values were represented as the mean (±SE), and the statistical significance was determined by using Independent-Samples T test with SPSS 16.0 software.

### Superoxide dismutase (SOD) and peroxidase (POD) activity assaying

The SOD and POD of each sample was measured from dissected hindgut using SOD Assay Kit-WST (Dojindo, Japan) and Quantitative Peroxide Assay Kits (Thermo, USA), respectively. Values were represented as the mean (±SE), and the statistical significance was determined by using Independent-Samples T test with SPSS 16.0 software.

## Additional Information

**How to cite this article**: Tan, S.-q. *et al.* The mechanism for microsporidian parasite suppression of the hindgut bacteria of the migratory locust *Locusta migratoria manilensis. Sci. Rep.* **5**, 17365; doi: 10.1038/srep17365 (2015).

## Supplementary Material

Supplementary Information

## Figures and Tables

**Table 1:** The number and length of valid sequences of infected locusts (IL) and uninfected control locusts (CL).

Sample	Sequences	Bases (bp)	Average Length (bp)
IL	22076	8739742	395.89
CL	27439	10862281	395.87

**Table 2:** Diversity indices of bacteria in the hindguts of Infected Locusts (IL) and uninfected control locusts (CL).

Sample ID	0.97				
	OTU	Ace	Chao	Shannon	Simpson
IL	20	22 (20,32)	21 (20,31)	0.84 (0.82,0.85)	0.5672 (0.5603,0.5741)
CL	27	27 (27,31)	27 (27,0)	1.28 (1.27,1.29)	0.3376 (0.3345,0.3407)

OTU = Operational Taxonomic Units of bacteria present in the hindgut.
