# Supplementary material for: The mechanism for microsporidian parasite suppression of the hindgut bacteria of the migratory locust Locusta migratoria manilensis
Source: Sci Rep. 2015 Nov 27;5:17365. doi: 10.1038/srep17365 (PMC4661595; doi:10.1038/srep17365)
Supplement: Supplementary Information [file srep17365-s1.doc]

**The mechanism for microsporidian parasite suppression of the hindgut bacteria of the migratory locust *Locusta migratoria manilensis***

**Shu-qian Tan, Kai-qi Zhang, Hong-xing Chen, Yang Ge &** **Wang-peng Shi***

Corresponding author: Wang-peng Shi

Department of Entomology, China Agricultural University, Beijing, China

Postal address: Yuanmingyuan Xilu NO.2, College of Agronomy and Biotechnology, CAU. Beijing, 100193 China

e-mail: [wpshi@cau.edu.cn](mailto:wpshi@cau.edu.cn)

Work telephone number: 86-10-62733505/3569

**Supplementary Information**

**Tables**

Table S1 Primers for quantitative RT -PCR validation of selected differentially expressed unigenes related to *Peroxidase* and *attacin* and primers for 16s rRNA cloning

| Genes | Sequence (5’🡪3’) |
| --- | --- |
| *β-actin* | CGAAACCTTTAATACCCCAG |
|  | CCATCACCAGAATCCAACAC |
| *attacin* | GTGCTCCTCGTCGTTCTGA |
|  | CCCACGCCTTTCTCTCTGT |
| *Peroxidase* ^a^ | CATGGAGTTCAGGAAGTTTTGC |
|  | CATGGAGTTCAGGAAGTTTTGC |
| 16s rRNA | 515F: GTGCCAGCMGCCGCGG |
|  | 907R: CCGTCAATTCMTTTRAGTTT |

^a^ *Peroxidase* sequence was obtained from transcriptome sequencing data published by Wangpeng Shi (Wangpeng Shi et al., 2014) and its unigene ID is comp134436_c1_seq1.

Table S2 Community structure of the lab colony (species level)

| Taxon (species) | OUT *No.* | [ratio](app:ds:ratio) in [community](app:ds:community) | |
| --- | --- | --- | --- |
|  |  | CL | IL |
| *Advenella sp. KT106* | OUT 16 | 0.000497 | 0 |
| *Corynebacterium sp. WA7* | OUT 10 | 0.000153 | 0.001866 |
| *Myroides profundi* | OUT 9 | 0.000115 | 4.66E-05 |
| *Myroides sp. SCU-B1722* | OUT 13 | 0.000267 | 0 |
| *Ochrobactrum pseudogrignonense* | OUT 18 | 0.000267 | 0 |
| *Pectobacterium carotovorum* | OUT 22 | 0.002675 | 0.000187 |
| *Pseudomonas chlororaphis group* | OUT 23 | 0.00107 | 4.66E-05 |
| *Pseudomonas sp. HN-2* | OUT 14 | 0.000688 | 0 |
| *Psychrobacter cryohalolentis* | OUT 21 | 0.000382 | 9.33E-05 |
| *Raoultella (Klebsiella) terrigena* | OUT 17 | 0.432048 | 0.195607 |
| *Sphingobacterium shayense* | OUT 15 | 0.000229 | 0 |
| *Sphingobacterium sp. ML3W* | OUT 20 | 0.000535 | 0 |
| *Stenotrophomonas sp. TM16* | OUT 8 | 0.000458 | 0 |
| *Tricholoma matsutake* | OUT 19 | 0.004126 | 9.33E-05 |
| *Bacterium 1158* | OUT 11 | 0.000573 | 0.00014 |

CL= control locusts, IL = infected locusts.

Table S3 Diversity indices of bacteria in the hindguts of wild locusts (WL).

| Sample ID | 0.97 | | | | |
| --- | --- | --- | --- | --- | --- |
|  | OTU | Ace | Chao | Shannon | Simpson |
| WL | 50 | 50 (50,5) | 50 (50,50) | 2.41 (2.39,2.43) | 0.1267 (0.1246,0.1287) |

OTU = Operational Taxonomic Units of bacteria present in the hindgut

Table S4 Community structure of wild locusts (species level)

| Taxon (species) | OUT *No.* | [ratio](app:ds:ratio) in [community](app:ds:community) |
| --- | --- | --- |
| *Bacteroidetes bacterium ALI-INI1* | OUT 12 | 0.003172 |
| *Chryseobacterium haifense* | OUT 13 | 0.000498 |
| *Chryseobacterium jejuense* | OUT 28 | 0.000249 |
| *Chryseobacterium sp. T72F.09.LHR.H.Kidney.D* | OUT 15 | 0.000808 |
| *Empedobacter brevis* | OUT 22 | 0.000435 |
| *Epilithonimonas sp. NSG18* | OUT 19 | 0.000373 |
| *Flavobacterium sp. T14L.07.B.CHS.SRW.W.Kidney.D* | OUT 14 | 0.002488 |
| *Lactococcus lactis* | OUT 50 | 0.057711 |
| *Leuconostoc mesenteroides* | OUT 45 | 0.002425 |
| *Myroides profundi* | OUT 23 | 0.019963 |
| *Omocestus rufipes* | OUT 39 | 0.003358 |
| *Pseudomonas putida group* | OUT 46 | 0.000995 |
| *Pseudomonas sp. BSTT44* | OUT 40 | 0.104602 |
| *Pseudoxanthomonas sp. DR 4-09* | OUT 42 | 0.000187 |
| *Psychrobacter cibarius* | OUT 29 | 0.025435 |
| *Psychrobacter sp. ST4(2013)* | OUT 21 | 0.002488 |
| *Spermatophyta* | OUT 27 | 0.020833 |
| *Sphingobacterium sp. CV4* | OUT 38 | 0.000746 |
| *Stenotrophomonas sp. B+7* | OUT 17 | 0.000311 |
| *Streptococcus salivarius* | OUT 41 | 0.001555 |
| *Yersinia ruckeri* | OUT 48 | 0.002799 |

**Figures**


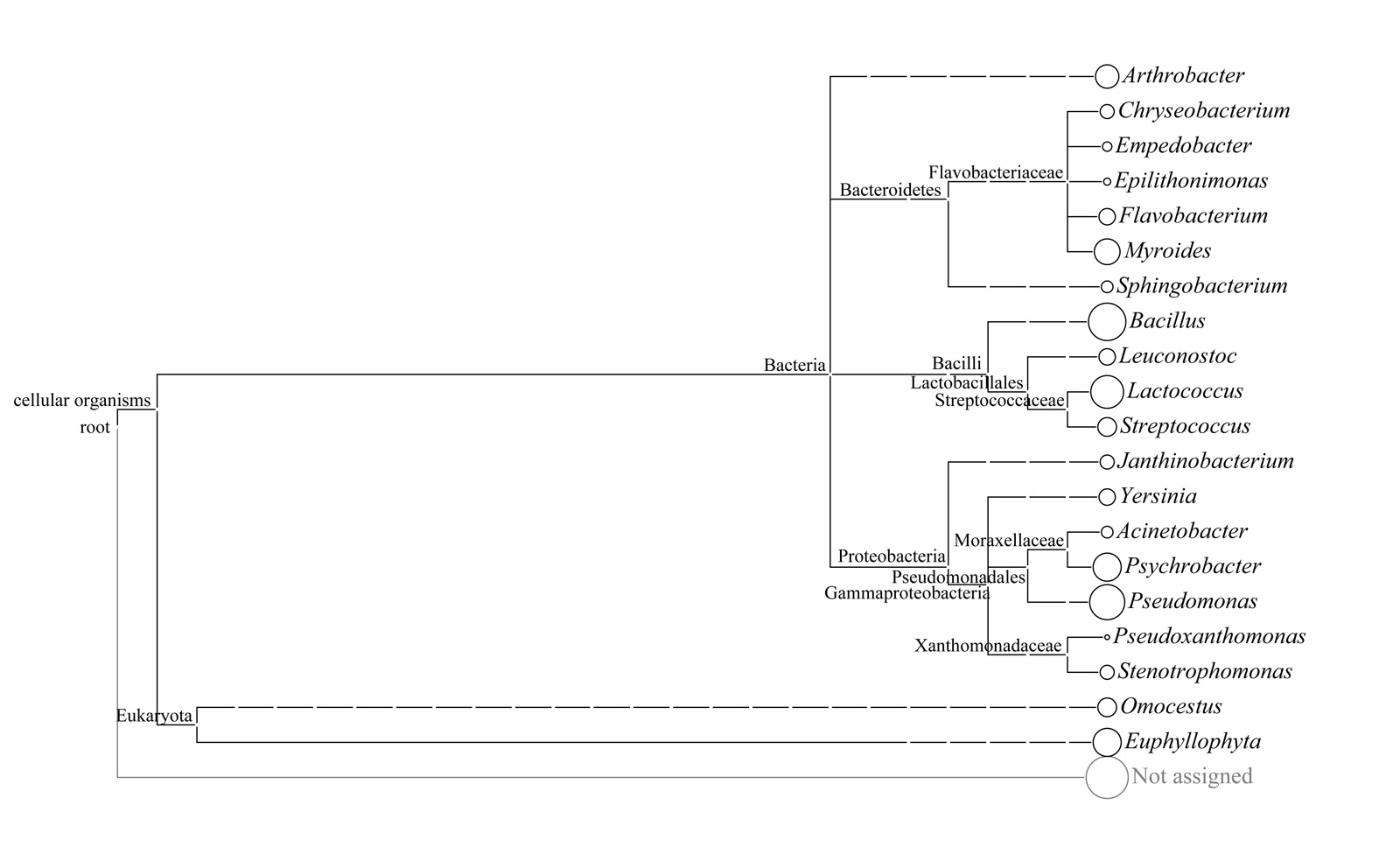


Fig. S1 Bacterial genera identified in the WL (wild locusts) [hindgut](app:ds:hindgut). Bacteria were classified according to branching in the 16S rRNA sequence phylogenetic tree. Sequences assignment results at the genus level. *Omocestus* and *Euphyllophyta* are two genera of fungus. Many other bacteria were not identified were included in “Not assigned”.


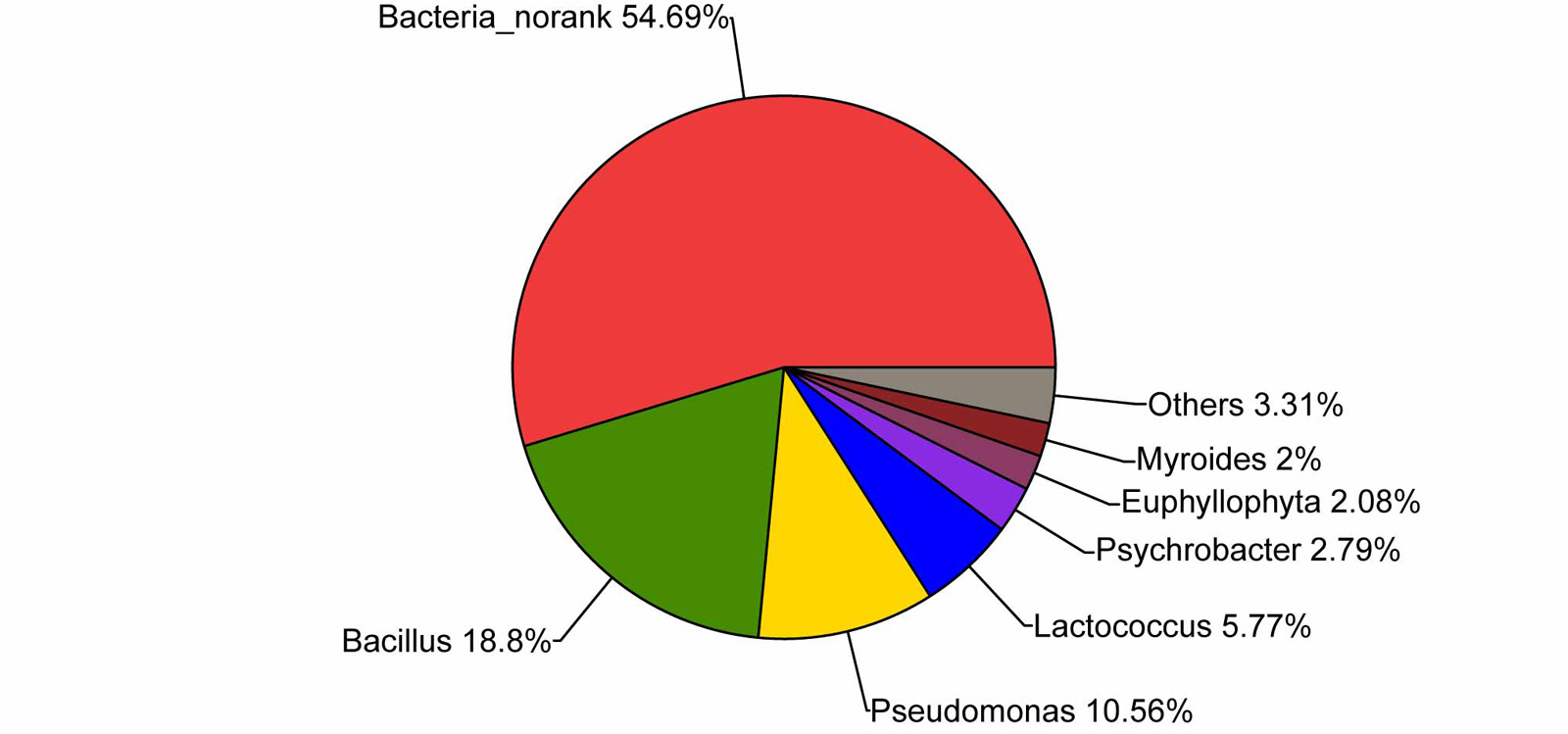


Fig. S2 Relative abundance of genus level classified for wild locusts. Many genera whose abundance were less than 1% were included in “Others”.
